# The Public Health Challenge of Consumer Non-Compliance to Toy Product Recalls and Proposed Solutions

**DOI:** 10.3390/ijerph15030540

**Published:** 2018-03-17

**Authors:** Xiayang Yu, David C. Schwebel

**Affiliations:** Department of Psychology, University of Alabama at Birmingham, Birmingham, AL 35294, USA; schwebel@uab.edu

**Keywords:** injury prevention, child health, health policy, health promotion, behavioral theories

## Abstract

This paper addresses the public health issue of toy product recalls in the United States, an under-addressed topic in scholarly literature, yet highly relevant to the prevention of pediatric injuries. Toy-related injuries led to 274,000 emergency room visits and seven fatalities in 2016 in the United States, and toy-related injury rates have remained stable over the last five years despite declining incidences of recalls. While dangerous toys not being recalled and the misuse of “safe” products are possible contributing factors, consumer non-response to recall notices also contributes to unintentional child injury from dangerous toys. We discuss the process of recalling toys, and the role of the U.S. Consumer Product Safety Commission in that process. We also review potential factors behind significant consumer non-response to recall notifications, citing economic and psychological theories as explanations for the actions of multiple stakeholders in the recall process. We close by proposing reforms at the regulatory, consumer, and retailer levels that might boost compliance with recall notifications and ultimately reduce injury morbidity and mortality.

## 1. Introduction

Toys and play greatly impact children’s cognitive, social and intellectual development [1,2]. For example, construction blocks, board games, and puzzles foster children’s spatial ability, which is crucial for success in the science, technology, engineering, and mathematics (STEM) fields [3]. Toys also cultivate interpersonal skills and may even have therapeutic effects on children with disabilities [4].

**Figure 1:**
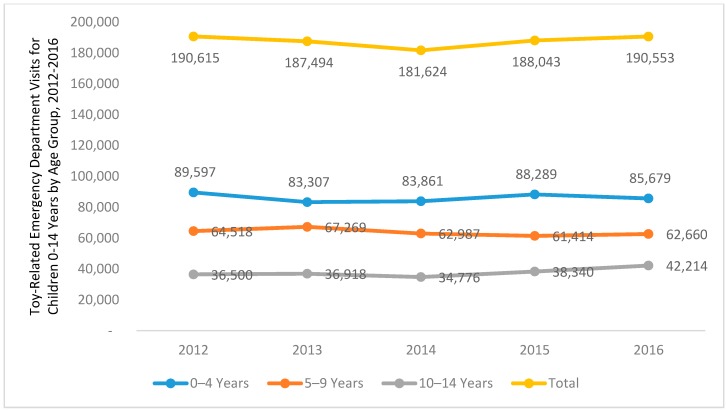
Toy-related emergency department visits for children 0–14 years by age group, 2012–2016. **S**ource: National Electronic Injury Surveillance System (NEISS), United States Consumer Product Safety Commission [5].

Nonetheless, toys are not without drawbacks. Playing with certain toys can result in serious injury. Figure 1 illustrates data from the National Electronic Injury Surveillance System (NEISS) database concerning toy-related emergency department visits from 2012 through 2016 in the United States [5]. In 2016, there were 274,000 emergency department visits in the U.S. related to toys, of which 191,000 (70%) were for children under 15 years of age [5]. As shown, the highest rate of injury was among the youngest children, ages 0–4 years, but substantial numbers of injuries occur throughout child development.

NEISS data suggest that, in line with developmental psychology research suggesting greater activity level and risk-taking in boys [6], males account for 61% of total toy-related injuries [5]. For children under 15 years of age, injuries are predominantly to the head and face region (53% of total) and laceration is the most common injury type (25% of total) [5]. Seven deaths were attributed to toys in 2016 [7]. The product types causing the most child injuries in the US include riding toys (23%), toy balls (10%), and toy vehicles (6%) [7]. 

One other point about toy-related injuries is notable: as illustrated in Figure 1, the annual incidence of toy-related injuries remained stable from 2012 to 2016 [5]. This trend is likely influenced by a wide range of factors that are resistant to behavioral change, including toy misuse (e.g., children playing with age-inappropriate toys or playing with toys in ways that they were not intended for) [8], inadequate caregiver supervision [9,10], injury by non-defective toys (e.g., collision with motor vehicle while on a scooter), and injury by recalled toys. Additional data and research are needed to ascertain the relative contribution of each in causing injuries, but we focus at present on the last factor, injury by recalled toys. 

Specifically, we propose that consumers have insufficient awareness of and response to toy recalls and that this contributes to toy-related injury rates in the U.S. In discussing this public health risk, we briefly overview the regulatory agency, the U.S. Consumer Product Safety Commission (CPSC), review current toy safety regulations in the U.S., and discuss toy recalls and impediments to the recall process. We close with proposed recommendations to multiple stakeholders in the recall process that might improve the dissemination of information regarding recalled toys and parental response to toy recalls. 

To perform our analysis, we utilized the academic journal search engine Scopus, entering the keywords “toy injury” and “product recall” to retrieve relevant articles. Our literature review also involved reading governmental websites such as CPSC.gov. Data collection involved querying the National Electronic Injury Surveillance System (NEISS) for the 0–14 years age group. Lastly, store visits to top toy retailers in the authors’ area of residence were completed to assess their store-based recall protocol from a consumer perspective.

### 1.1. The CPSC

The CPSC was created by Congress in 1972 to protect the public against “unreasonable risks of injuries associated with consumer products” [11]. Products under the CPSC’s purview include toys as well as other consumer goods used in the home, sports, recreation, and schools. To carry out its mandate, the CPSC prevents the sale of hazardous items and recalls products on the market that have caused or are likely to cause injury. 

Despite its jurisdiction over 15,000 product types, the CPSC has only about 500 employees [11], and therefore must balance proactive and reactive actions. This strategy creates potential for inadequate oversight [12]. From the proactive perspective, over the past four years, the CPSC has worked with U.S. Customs and Border Protection (CBP) to embargo over eight million units of 4500 different types of children’s toys and children’s products due to safety concerns and violations of federal safety standards [13]. Nonetheless, with 3000 to 5000 new toys introduced annually, proactive efforts cannot possibly be exhaustive and not all products are screened or inspected prior to sale to the public [14]. Consequently, the CPSC also engages in reactive actions, responding to approximately 400,000 consumer complaint calls per year, which translates to about 800 complaints per employee per year from citizens who purchased potentially defective products [15]. As a data-driven agency with limited resources, the CPSC states that it is only able to investigate products with higher volumes of consumer complaints [11]. For instance, IKEA’s (proper word and capitalization for the store) MALM (proper word and capitalization for the product) bedroom series dresser caused two fatalities of young children before a recall program was launched [16].

### 1.2. The Recall Process

**Figure 2:**
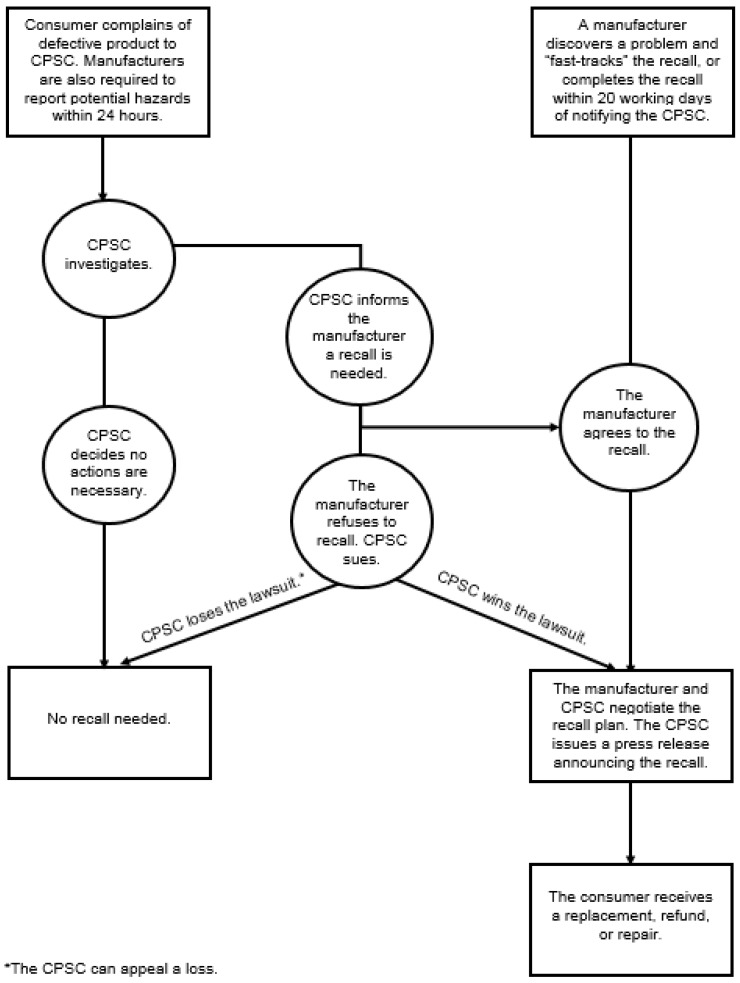
The U.S. Consumer Product Safety Commission (CPSC) recall process.

Figure 2 offers a high-level representation of the product recall process at the CPSC. Upon receiving sufficient complaints from consumers or self-reports from manufacturers about a hazardous item, the CPSC investigates the merits of the claims and then determines whether to issue a recall. If the CPSC recalls an item, manufacturers can contest the decision, although that situation has not occurred since 2001. In most cases today, voluntary company recalls are announced, often after manufacturer discussion with CPSC officials [17]. As the recall occurs, the CPSC issues a news release to the public, alerting consumers who can either return, replace, or repair the hazardous item, depending on the remedy specified by the manufacturer.

## 2. Poor Response Rates to Recalls

The number of CPSC recalls declined substantially between fiscal years 2008 and 2016, from 172 to 24 [18]. The agency attributes the downward trend to the institutionalization of additional safeguards: lowering acceptable lead and phthalate limits, setting certain mandatory standards for toys, and working with CBP to monitor shipments arriving at U.S. ports [13]. On the other hand, toy-related injury rates remain at historical levels [5]. 

As discussed in our introductory remarks, several possible explanations likely co-exist to explain the paradox of declining toy recalls yet stable injury rates, including misuse of safe toys, inadequate caregiver supervision, and use of risky but not recalled items. We focus herein on another factor—consumer unawareness of and non-compliance to recalls. Data across industries point to strong consumer non-compliance with recall notices. The experience of the automotive industry suggests that consumers may overestimate responsiveness to recall notices: a survey of over 1100 Americans found that 87% of participants report complying with automotive recall notices most or all of the time, but data from the National Highway Traffic Safety Administration suggest that only 62% of recalled cars are repaired after multiple notifications to consumers [19]. By self-report, millennials are significantly less likely to respond to auto recalls, with 78% doing so compared to 91% of adults over 55 [19]. This estimate from the automotive industry may be overly sanguine, however, when compared to data from the toy industry. Kids in Danger reports that only 10% of children’s products recalled in 2012 were successfully remedied [20].

Why do consumers fail to return products that their government deems dangerous to their families? Multiple explanations exist. In more optimistic scenarios, recalled items are not being remedied as they have been outgrown and set aside. Another logical explanation is that logistics for manufacturers and retailers pose a major barrier [21]. For relatively inexpensive toys that are not registered with the manufacturer upon purchase, the identities of the consumers who own dangerous products may often be unknown to the retailer, the manufacturer, or the government. Thus, no one can easily locate and alert consumers when a hazard is discovered. 

In some cases, retailers can identify the consumers who purchased recalled products. This is particularly the case for online retailers, who possess electronic records of purchasers, including their email and postal addresses. However, online retailers vary widely in their policies on contacting consumers when recalls occur. Amazon proactively removes recalled items from its website and reaches out to buyers and sellers of recalled items. When Amazon discovered deficient solar eclipse glasses had been sold on its website, for example, consumers were contacted and refunded [22]. eBay, in contrast, forbids the sale of recalled items on its platform, but has not implemented any consistent or formal policy of contacting buyers of recalled items. 

Information concerning traditional “brick-and-mortar” retailers is less available. We searched for recall policies from Walmart and Toys “R” Us, for example, but could not locate anything on the retailers’ respective corporate websites. In more extreme cases, traditional retailers have been cited for laxity in monitoring their stores for recalled items. In August 2017, Home Depot paid a $5.7 million civil penalty to the CPSC for intentionally selling and distributing approximately 2816 recalled products between August 2012 and November 2016 [23]. 

**Figure 3:**
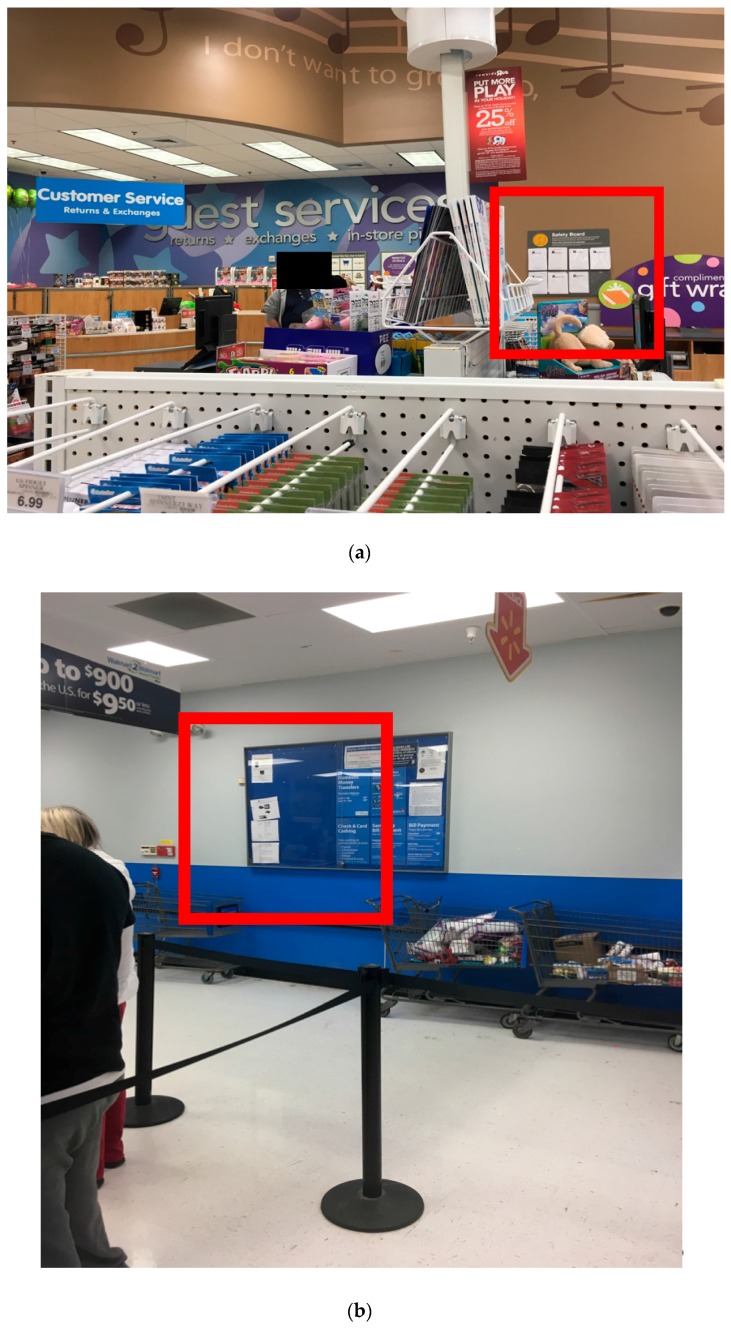
Where recall notices are found at local retailers. (**a**) The “Safety Board” at a major toy store in Birmingham, Alabama. Product names of recalled items are listed in a small font and no photos of the products are provided to help consumers identify items they might own. (**b**) Only two recall notices were posted in the customer service area at a major multi-product retailer in Birmingham, Alabama. The customer service area is located in a lightly-trafficked part of the store, away from where many consumers might view it.

Certain manufacturers and retailers have employed ineffective methods of notifying buyers of recalled items. For instance, the CPSC directs firms conducting the recall to notify customers on social media, a mandate that appears to be loosely enforced. In 2013, there were 63 recalls in which the manufacturers had Facebook profiles, but only one in nine (14%) of those cases was Facebook utilized to notify customers [20]. Major retailers also inadequately advertise recalls in their stores, as Figure 3 demonstrates in example photos from local retailers. While manufacturers and retailers may issue recalls for hazardous items to comply with the law, they benefit financially from non-response, which could explain why recall notices are hidden, announced only to the extent that legal compliance is achieved. This behavior likely contributes to low recall response rates and ultimately to increased child injury rates.

Desire to placate investors may also impact how companies execute recalls. A recall incident can impact the company’s market share, revenue of recalled products, stock price, purchase intent, and sales of other company products [24,25,26]. Research demonstrates that to preserve customer purchase intent, voluntary recall is the best option for established firms with positive media coverage, while “super-effort”, that is, going above and beyond with the recall effort, is recommended for firms with unknown or weak reputations [25]. Despite gaining customer loyalty, firms that employ a proactive recall approach are not rewarded by the financial markets [15], and thus firms might adopt a passive recall strategy at the expense of public safety. In one study, for example, the authors inferred that investors treat a proactive recall decision as a sign of major product liability that could create massive financial losses when calculating equity value [15].

Non-compliance with recalls is not solely a result of manufacturer and retailer decisions. The attitudes of consumers toward the recall process may also result in non-compliance. The Health Belief Model (HBM) [27], which has been validated to study why people make decisions that may undermine the health of themselves and their families, offers guidance. The HBM postulates that individuals select a prescribed health action if they feel the threat of illness/injury and consequences thereof are severe, the health action is beneficial, and the barriers to implementing the action are low [27]. By analogy, consumers may underestimate susceptibility to injury resulting from interacting with recalled items. They also may underestimate the benefits of responding to a recall and overestimate the barriers to doing so. Just as smokers struggle with quitting and obese individuals struggle with dieting, consumers may struggle to find the time, energy, and motivation to respond to toy recalls even when they have the information that a toy they own is potentially dangerous to their family.

## 3. Recommendations

In the interest of public safety, action by multiple stakeholders is recommended to improve the toy recall process. Below we offer recommendations for each group of stakeholders—retailers, consumers, and regulators. 

### 3.1. For Retailers

The example of Amazon’s successful recall of faulty solar eclipse glasses proves that online retailers can readily notify consumers of hazardous products. Congress and the CPSC can, in conjunction, enact and enforce policies that oblige online retailers to email consumers when items in their purchase history have been recalled. For brick-and-mortar retailers who cannot easily identify purchasers of particular products, broad dissemination of notifications should be conducted in stores. Photos and descriptions of recalled items could be placed in prominent locations in stores, including entrances and checkout areas. Also, notifications could be printed on receipts and regular weekly ads.

### 3.2. For Consumers

To minimize risk of injury, consumers should be vigilant regarding child safety and recalled items. To preserve child safety, caregiver monitoring and supervision of young children is essential [10]. Supervision and monitoring serve as buffers to child injuries, both by actively intervening to prevent children from engaging in risky behaviors and by increasing children’s self-regulation [28]. Policy statements from organizations such as the American Academy of Pediatrics (AAP) guide caregivers on recommended supervision levels for various age groups and activities. As an example, the AAP recommends that children under 10 years of age should not use skateboards without close adult supervision [29].

Although a challenging task, ideally caregivers will also stay aware of toys they own that may be recalled. One resource to support this vigilance is Recalls.gov, a website that amalgamates recall notices from six federal agencies with jurisdictions ranging from toys to food and creates a “one-stop shop” for consumers, simplifying the task of staying abreast of recall information. Consumers can search for all active recalls on the site, as well as elect to receive daily emails from agencies on new recalls.

Ultimately, consumer behavior is a health behavior driven by motivations that parallel other health-related behaviors such as nutrition, exercise, safe sex, and medication adherence. As such, behavioral researchers should conduct research to identify means of motivating affected consumers to take action. This research—and the health interventions that are implemented—might parallel other health behavior research and can borrow lessons learned from more established behavioral medicine literatures.

### 3.3. For Regulators

For regulators, efforts could ameliorate both the front- and back-end of the toy recall process. Not only should the volume of harmful toys reaching consumers be reduced (the front-end), but those items must also be removed from the market more effectively (the back-end). 

On the front-end, the CPSC can increase staffing or utilize contractors to examine more incoming toy shipments for safety. As previously mentioned, while the number of toy recalls has declined, toy-related injuries have not, implying the possibility that some dangerous items are being missed. On the back-end, increased staffing also means more consumer complaints could be investigated promptly, reducing the number of injuries that take place prior to the CPSC taking action. To achieve both these recommendations, budget appropriation from Congress is needed. With the CPSC costing taxpayers only $125 million per annum [30], it could be argued that an increase in funding is reasonable. We also suggest that the CPSC should invest in technology, in particular smartphone technology, to more quickly and adeptly reach today’s consumers. Smartphones could be a valuable vehicle to deliver product recall notices to consumers, as 77% of American adults have a smartphone and ownership reaches 92% among the prime age of young parents, between 18 and 29 years [31]. A mobile app that notifies consumers of recalled items via banners or alerts on the home screen could be developed and freely distributed to the public. Additionally, customers who use mobile pay could be notified by those apps when items in their purchase history are recalled.

Regulations on data quality could also assist with injury prevention efforts surrounding toy recalls. Brief, incomplete, or inaccurate reports provided by hospital-based coders can invalidate NEISS data, making it difficult to use properly in research or prevention [32]. Adding more detailed coding of child injury causes, though labor-intensive and therefore expensive, could also improve prevention efforts to the point that it is financially justified. We were unable, for example, to determine from the NEISS data whether toy-related injuries were caused by recalled toys or some other cause. With more detailed coding of injury cause factors, limited resources for prevention and intervention could be targeted to the most urgent needs. 

## 4. Limitations and Future Directions

Our study aims to explain consumer non-response rates to toy product recalls. A limitation is our lack of experimentation to justify our thesis; therefore empirical research on the topic is recommended. From a basic science perspective, for example, it would be worthwhile to investigate the differences between respondents and non-respondents to recall notices, employing constructs of the Health Belief Model to guide research designs. From an applied perspective, randomized controlled trials might examine the feasibility of interventions to increase recall response rates, including smartphone-based recall alert apps. 

## 5. Conclusions

The toy recall process suffers from extraordinarily low response rates. We suggested that this could reflect difficulty reaching consumers, misaligned incentives of companies handling recalls, and inaction on the part of consumers. In the interest of child safety, the recall process would strongly benefit from reforms and additional resources. To that end, we proposed changes at the retailer, consumer, and regulatory levels. Adoption of these recommendations could help consumers stay abreast of product recalls and ultimately reduce unintentional injuries to America’s children.
